# Does stigmatization moderate the association between intention and implementation of learned prevention-strategies at work after a depressive episode? – a cross-sectional pilot study

**DOI:** 10.1186/s12995-019-0246-9

**Published:** 2019-11-05

**Authors:** Petra Maria Gaum, Franziska Brey, Thomas Kraus, Jessica Lang

**Affiliations:** Institute for Occupational, Social and Environmental Medicine, University Hospital RWTH Aachen, Pauwelsstraße 30, 52074 Aachen, Germany

**Keywords:** Depression, Return to work, Social stigma, Intention, Implementation, Prevention, Humans

## Abstract

**Background:**

A depressive episode is a frequent reason for production loss due to long periods of absence at work. To maintain work ability after depression, affected employees need to implement learned coping strategies from interventions at work. Based on the theory of planned behavior, this paper examines how stigmatization relates to the implementation of the learned strategies at the workplace. Further, differences between employees with single or recurrent depressive episode were considered.

**Methods:**

Data of an online survey from 112 participants who returned to work after sick leave because of a depressive episode were analyzed [men = 28 (25%); Age: mean = 42.3, SD = 10.9]. The strategies learned were asked openly, intention and implementation with a questionnaire based on the theory of planned behavior and stigmatization with an adapted version of the German inventory of subjective stigma experience.

**Results:**

Intention is positively (β = .46, *p* < .001) and anticipated (β = −.18, *p* = .052) and experienced stigmatization not (β = −.11, *p* = .27) correlated with implementation. Only anticipated stigmatization moderates the association between intention and implementation (β = .26, *p* = .003). If individuals report a high intention to implement the learned strategies, stigmatization has no influence on implementation. Under low intention, stigmatization leads to less implementation. Participants with recurrent depressive episodes report higher anticipated stigmatization than participants with a single episode.

**Conclusion:**

When employees return to work after a depressive episode, it is important to address anticipated stigmatization and to develop an organizational culture that helps them to reduce their fear of stigmatization and strengthens their work ability via implementing learned prevention-strategies. The reduction of patient’s anticipated stigmatization should already be considered in the therapy and reduced in cooperation with occupational physicians.

## Background

The prevalence rate of depression has been steadily increasing in recent years, especially in industrial nations such as in the USA, Belgium or in Germany [1]. Depression is a common reason for sickness absence and early retirement [2], which often means a social decline for those affected and economic costs for companies and health systems [3]. In the USA, the projected total annual economic loss due to major depression is estimated at $36.6 billion [4]. A problem arises in particular because of the chronification of depression that is characterized by recurrent depressive episodes. A total of 66% experience a recurrence [5]. The average age of onset of depression is around the age of 30 and thus relatively at the beginning of working life [6]. Of affected employees 85.4% return to paid work after a depressive episode [7]. However, within 1 year employees with depression have a higher probability to be unable to work again due to their illness than employees with a chronic somatic disease [8]. This study focusses how the work ability of employees who return after a depressive episode can be maintained in the long term.

In the context of therapy and rehabilitation, patients learn prevention-strategies in interventions in order to alleviate their symptoms and maintain their state of health. Hallgreen et al. [9] report, that common interventions such as psychotherapy or pharmacotherapy have a positive effect on the work ability of depressive workers, but these effects were very small. In a review, Furlan et al. [10] compared 12 studies with interventions to maintain work ability in depression. The interventions considered ranged from behavioral therapy to interventions for stress reduction. However, all the interventions examined also had very little effectiveness in terms of reducing sickness absence days or improving health behavior at the workplace. A meta-analysis by Martin et al. [11] showed positive effects of work-related interventions on symptoms of depression, but these effects were very weak. The authors discuss that for many depressive employees it is difficult to implement the behaviors and learned prevention-strategies in everyday working life. The implementation of the learned prevention-strategies is the precondition for their effectiveness. The theory of planned behavior [12] and the associated intention-behavior gap [13] could provide an explanation for this low implementation of learned prevention-strategies at work.

Ajzen [12] describes in his *theory of planned behavior* that the intention to show behavior is positively correlated with the behavior itself. The stronger the intention to show a behavior, the higher is the probability that the behavior will be implemented. Applied to learned prevention-strategies, this would mean that a high intention to implement the strategies of affected employees will result in a higher probability to implement these prevention-strategies. However, the literature also describes that the intention does not necessarily lead to behavior implementation. If a behavior is not shown despite of a high intention, one speaks about the intention-behavior gap [13]. This intention-behavior gap occurs also in the implementation of health-relevant behavior [14]. As possible reasons for health-related intention-behavior gap a failure to monitor the progress or bad habits were discussed [13]. Especially in mental illness, stigmatization plays a major role [3]. This study examines whether stigmatization has an influence on the relationship between intention and implementation and whether it represents a possible reason for the intention-behavior gap in the implementation of learned prevention-strategies after depressive episode.

Humans build their social identity by attributing characteristics towards themselves or by characteristics that their social environment attributes to them. An „attribute that is deeply discrediting “is called stigma [15]. In our study we differentiate between anticipated (AS) and experienced stigmatization (ES). AS is defined as “the degree to which individuals expect that others will stigmatize them if they know about the concealable stigmatized identity” [16] and ES as “the individual’s perception of being stigmatized by others” [17].

In general, stigmatization has been associated with reduced job satisfaction in employees with chronic disease [18]. Stigmatization is also negatively associated with help-seeking behavior in patients with mental disorder [19]. AS from work colleagues is positively associated with stress and negatively with social support and quality of life [20]. Considering the topic of the present work, we want to examine the potential influence of stigmatization by individuals in the work environment (i.e. supervisors and colleagues) on the implementation of learned prevention-strategies of depressed employees.

A prior study report that the severity of a depressive episode is associated with the perception of stigmatization [21], but to the best of our knowledge, in no study the impact of the number of depressive episodes on the perception of stigmatization was considered. It might be that employees with a recurrent depressive episode have another perception of stigmatization than employees with the first depressive episode. Experiences of stigmatization during previous episodes may influence the anticipation and perception of stigmatization in following episodes. Further, it is possible that a second depressive episode can be interpreted as a loss of control. If an affected individual has the goal to prevent a recurrent depressive episode and implement behavior to reach this goal, an onset of a recurrent depressive episode may be interpreted as individual failure. Perceived control can influence the intention and implementation of behavior [12]. Therefore, complementary research questions are considered in the present study to investigate differences in the considered variables between participants with one and with recurrent depressive episode.

Specifically, the present pilot study examines three hypotheses (H) and three complementary research questions (RQ) to give a first view on the possible association of stigmatization when implementing prevention strategies. The first hypothesis examines whether there is a positive relationship between intention and implementation (H1) according to the theory of planned behavior. This means that a stronger intention is associated with a higher probability of implementing learned prevention-strategies. In the second hypothesis, the direct relationship between AS (H2a) and ES (H2b) with implementation is tested. We expect a negative association between both types of stigmatization and the implementation of learned strategies. Related to the intention-behavior gap, the third hypothesis examines that AS (H3a) and ES (H3b) moderate the relationship between intention and implementation of learned prevention-strategies. If stigmatization is low the postulated positive association between intention and implementation should be found. This positive association should be weaker in case of high stigmatization. Additionally, in the complementary research questions, we investigate whether there are differences in intention (RQ1), the implementation of learned strategies (RQ2) and the amount of AS (RQ3a) and ES (RQ3b) according to the number of depressive episodes.

## Methods

The aim of this study is to enhance the implementation of the learned strategies at the workplace to prevent a recurrence in employees with former depressive episode and how social stigmatization can influence this. The theory of planned behavior is used as theoretical frame for this study. Further, differences in intention, implementation and stigmatization between employees with single or recurrent depressive episode were considered. This study was approved by the local ethics commission of the Medical Faculty of RWTH Aachen University (EK 187/17). All participants provided informed consent prior to their participation.

### Design, setting and study population

The data for this cross-sectional study were collected from July the 11th to August the 22nd 2017 in Germany using an online questionnaire (SoSci Survey). The link was distributed via self-help groups, social media and the German Depression League. The link was also printed on flyers and displayed in offices of general practitioners, pharmacies and in a large university hospital. We were looking for participants who had been on sick leave due to a depressive episode before and already returned to work at the time of the study. A total of 398 participants clicked on the questionnaire, 146 participants dropped out and 187 participants completed the questionnaire. At the beginning of the questionnaire, all participants were openly asked about prevention-strategies they have learned during their therapy and rehabilitation to provide them with an anchor on which they can refer the further questions on intention and implementation to. Using the cut-off criteria of the depression scale of the patient health questionnaire (PHQ) [22], participants were categorized for a current depressive episode (yes/no). Participants who showed an acute depressive episode were excluded from all analyses (*n* = 74). Furthermore, participants were excluded who did not specify a prevention-strategy (*n* = 3), who worked despite the depressive episode (*n* = 11) or have not yet returned to work (*n* = 44). Also one participant of minor age was excluded. The final sample consists of 112 participants with 28 men (25.0%) and 84 women (75.0%) and a mean age of 42.3 years (SD = 10.9y).

### Variables

#### Intention and implementation

Items to measure intention and implementation were generated according to Francis et al. [23]. Three items were used for each; intention (e.g. “When I resumed work, I wanted to implement the strategies and change my behavior”) and implementation (e.g. “Now that I have been back to work for some time, I can say that I am implementing the strategies in my daily work”). All items had a seven-point Likert-response-scale ranged from “does not apply” to “perfectly apply”. Cronbach’s Alpha was .88 for intention and .86 for implementation. Mean values were calculated for both variables.

#### Stigmatization

Stigmatization was assessed with adapted items from the German inventory of subjective stigma experience (ISE; Inventar subjektiver Stigmaerfahrungen) [24]. We measured AS and ES with two items each and adapted the items to the working context. An example of the AS was “Do you think that your colleagues and your supervisor will think less of you if they would know that you have a mental illness” and for ES “Have you ever been teased, harassed or molested by one of your colleagues or your supervisor, because you have a mental illness?”. Each item had a four-point response scale from zero (“never”) to four (“almost always”). Cronbach’s alpha was .75 for AS and .84 for ES. The mean values of the items were used for both scales.

#### Depressiveness

Depressiveness was measured with the German version of the patient health questionnaire (PHQ-D [22];). The scale consists of 9 items considering the ICD-10 and DSM-IV criteria for depressive symptoms such as “Feeling down, depressed, or hopeless”. The participants had to specify how often they felt bothered by the respective symptoms during the last 2 weeks. The response scale ranged from zero (“not at all”) until three (“nearly every day”). Since depression can affect the perception of stigmatization [21], we created a sum scale of all items (Cronbach’s alpha = .74) and included it as control variable.

#### Additional information

As additional information, the current intake of antidepressants and other psycho-pharmaceuticals was asked for. Participants were also asked how long they were on sick leave during their last episode. To record the duration of the sick leave, the number of years, months and days of the last sick leave due to a depressive episode was asked. The time unit “days” was used for the duration of sick leave [(years * 364) + (months * 30) + days]. Participants were also asked on what date they returned to work after depressive episode. The date on which the questionnaire was answered was also entered. Then the difference was calculated from these two dates, which shows the time how long participants have been back at their workplace (in “months”).

### Statistical analyses

Hypotheses 1 and 2 were tested with linear regression. Implementation was used as criterion variable. Intention (H1), AS (H2a) and ES (H2b) were entered as predictor variables. The moderation hypotheses (H3a & 3b) were tested with the SPSS macro PROCESS by Hayes [25].

To answer the complementary research questions (RQ1, 2, 3a & 3b), two groups were created. The first group includes participants with the first episode (*n* = 25; 22.3%) and the second group participants with a recurrent depressive episode in their life (*n* = 84; 77.7%). Both groups were compared with an analysis of variance (ANOVA). Intention, implementation and the two types of stigmatization were used as dependent variables. Except for the ES, the Levene-test was not significant. This means that the variances in intention, implementation and AS were homogeneous in both groups.

All analyses were performed with SPSS 25 for Windows [26]. The level of significance was *p* < .05 for two-tailed *p*-values. Statistical power analyses were performed for significant results with G*Power 3.1.9.2 [27]. Nevertheless, a 95% confidence intervals were generated with bootstrapping (*N* = 1000), because of the small sample size and to strengthen the results of the multiple regression analyses.

## Results

The study population show a relatively high mean intention and implementation and a relatively low mean stigmatization. A detailed description of the study population is given in Table 1. The Spearman’s rank correlation coefficients of the associations between the relevant variables are mentioned in Table 2. Because of significant correlation with the dependent variable, all analyzes were controlled for depressiveness. The duration of sickness absence and the time since back were not correlated with intention, implementation or stigmatization. Therefore, it was not controlled for the duration of sickness absence and time since back.

**Table 1:** Description of study population (*N* = 112)

	Scale	Mean	Standard deviation	Median	Range
Intention	1–7	5.8	1.3	6.0	1–7
Implementation	1–7	5.2	1.3	5.3	1–7
Anticipated stigmatization	1–5	2.6	1.1	2.5	1–5
Experienced Stigmatization	1–5	1.8	1.1	1.5	1–5
depressiveness	0–27	7.2	3.8	7.0	0–20
# of depressive episodes	1–10 (and more)	3.7	2.7	3.0	1–10
Time since back^a^	–	39.7	55.3	23.0	1–490
Duration of the sickness absence^b^	–	435.1	515.0	210.0	10–2920

# = number, ^a^in months, ^b^in days

**Table 2:** Spearman’s rank correlation coefficients (*N* = 112)

	1)	2)	3)	4)	5)	6)	7)	8)	9)	10)
1) Age	–									
2) Gender	−.04	–								
3) Intention	.02	.05	–							
4) Implementation	.03	−.06	.28**	–						
5) Anticipated stigmatization	−.06	.17+	−.13	−.23*	–					
6) Experienced stigmatization	−.07	.19*	−.14	−.24*	.69**	–				
7) Depressiveness	−.02	.23*	.03	−.33**	.19*	.30**	–			
8) Psychopharmacy	.03	.07	.04	−.18+	.08	.10	.02	–		
9) # of depressive episodes	.03	.12	.07	−.13	.22*	.30**	.31**	.27**	–	
10) Time since back^a^	.18+	−.07	.04	.18+	−.15	−.14	−.26**	−.16+	−.16	
11) Duration of sickness absence^b^	.15	−.06	.03	.18+	−.09	−.07	.01	.09	.23*	.13

# = number; ^a^in months; ^b^in days; *p*-value (significance; two-tailed): + = .05 < *p*-value <.1; * *p*-value <.05; ** *p*-value <.01

**Fig. 1:**
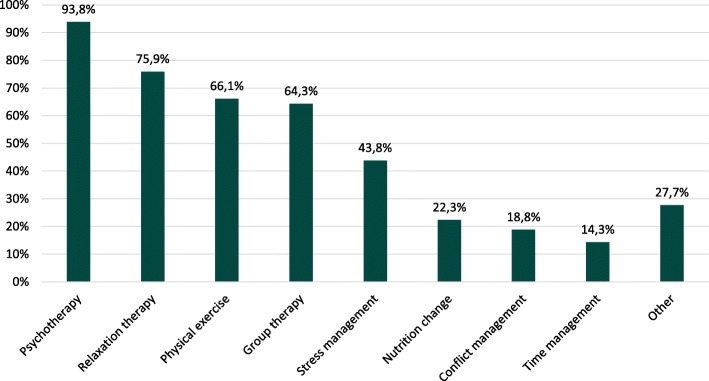
Relative frequency of reported interventions

Of the specifically asked interventions, a mean amount of 3.84 (SD = 1.9) participated interventions were reported. Figure 1 illustrates the mentioned interventions. Approximately 27% report “Other” interventions e.g. music therapy or occupational therapy. According to open asked learned prevention-strategies, mostly mindfulness (17.9%) and taking breaks (17.0%) were mentioned.

In the first hypothesis, the direct association between intention and implementation of learned prevention-strategies was investigated. The regression analysis shows a significant positive association [β = .46, *t* (109) = − 5.64, *p* < .001, 95%-CI: .18–.67]. The overall model was also significant [*F* (2,109) = 22.13, *p* < .001] and intention explained 18.7% of the variance of implementation. Hypothesis 1 could be confirmed.

In the second set of hypotheses we expected a negative association between AS (H2a) and ES (H2b) with the implementation of learned prevention-strategies. There was no significant correlation between AS and implementation [β = −.18, *t* (109) = − 1.97, *p* = .052, 95%-CI: −.43 – -.02]. For ES no significant association with implementation could be found, either [β = −.11, *t* (109) = − 1.11, *p* = .27, 95%CI: − 42 – .09]. Neither hypothesis 2a nor hypothesis 2b could be confirmed.

**Fig. 2:**
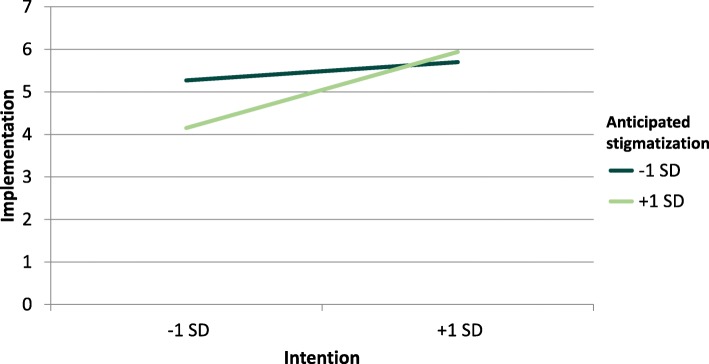
Simple slopes for standard deviation (SD) for low and high anticipated stigmatization

Within the moderator hypotheses, it is postulated that AS (H3a) and ES (H3b) moderate the positive relationship between intention and implementation. We found a significant interaction for AS (Table 3). The overall model was significant [*R*^2^ = .601, *F* (4,107) = 15.13, *p* < .001]. The moderation is illustrated in Fig. 2. For ES the overall model was significant [*R*^2^ = .558, *F* (4,107) = 12.11, *p* < .001], but the interaction term was not (Table 3). Thus, only hypothesis 3a could be confirmed.

**Table 3:** Results of the moderation analyses

	*B*	S.E.	β	*t*	*p*	Δ*R*^2^	95% CI (bootstrapping)
Anticipated stigmatization							
Intention	−0.18	0.22	.47	6.05	<.001		.32–.63
Anticipated stigmatization	− 1.57	0.48	−.19	−2.47	.02		−.34 – -.04
Intention*anticipated stigmatization	**0.24**	**0.08**	**.26**	**3.00**	**.003**	**.054**	**.09–.44**
Experienced stigmatization							
Intention	0.21	0.18	.49	5.85	<.001		.32–.66
Experienced stigmatization	−0.94	0.56	−.15	−1.62	.11		−.33–.03
Intention*experienced stigmatization	0.14	0.09	.16	1.50	.13	.014	−.05–.36

Controlled for depressiveness. B = unstandardized regression coefficient, S.E. = standard error, β = standardized regression coefficient, t = t-value, p = *p*-value (significance), ΔR^2^ = change in R^2^ (explained variance by interaction term), CI = confidence interval generated with bootstrapping (*N* = 1000). Significant result in bold

In the complementary research questions, we investigated whether participants with single episode differ from participants with recurrent depressive episodes in intention (RQ1), implementation (RQ2) and AS (RQ3a) and ES (RQ3b). Between the groups there was only in AS and ES a significant difference (see Table 4).

**Table 4:** ANOVA of participants with single (*n* = 25) and recurrent depressive episodes (*n* = 87)

Dependent variable	Scale	*M* (*SD*)		*F*	*df*	*p*	η^2^
		Single episode	> 1 episode				
RQ1: Intention	1–7	6.07 (0.90)	5.72 (1.39)	1.74	1	.19	.016
RQ2: Implementation	1–7	5.79 (1.17)	5.08 (1.28)	3.78	1	.06	.033
RQ3a: anticipated stigmatization	**1–5**	**1.94 (0.87)**	**2.78 (1.09)**	**10.07**	**1**	**.002**	**.085**
RQ3b: experienced stigmatization	**1–5**	**1.34 (0.86)**	**1.97 (1.06)**	**4.58**	**1**	**.035**	**.040**

Controlled for time since back and depressiveness; *M* mean, *SD* standard deviation, *F* F-value, *df* degrees of freedom, *p p*-value (significance), *η*^*2*^ explained variance, *RQ* research question. Significant results in bold

## Discussion

Employees who returned to work after a depressive episode report that the implementation of learned strategies from interventions is difficult at work, although they report a high intention to apply their skills [11]. This study investigated whether this intention-behavior gap can be explained by AS and ES.

The results show a positive correlation between intention and the implementation of learned prevention-strategies. This reflects the postulated association from the theory of planned behavior [12]. Intention to show a behavior is developed when a person wants to achieve a set goal (e.g. “I don’t want to get a new depressive episode”). During rehabilitation and therapy, patients learn strategies to prevent a new depressive episode. To achieve the goal, these learned strategies must be implemented. However, stigmatization is not associated with implementation when controlling for depressiveness. Studies have already shown that the perception of stigma is stronger in people with depression [21]. Depressiveness and stigmatization also correlate in this study. This multicollinearity could be one reason why the effect of stigmatization on implementation is not significant when controlling for depressiveness.

Only AS moderates the association between intention and implementation. Individuals reporting high AS, show less implementation under low intention than individuals not suffering from high AS. This means that AS in employees with low intentions leads to less implementation of the learned prevention-strategies. One reason, why only the AS has a moderating effect, could be the individual stereotypes and prejudices of the depressive employees. There are several levels of stigmatization [28]. The first level is the cognitive level concerning stereotypes and includes the knowledge we have about the group of depressive humans. Individuals generally attribute specific symptoms to depression like fatigue or listless. This knowledge leads to prejudice (e.g. “depressive workers are not efficient”) and thus to the second emotional level, prejudices. Prejudices are negative characteristics which are assigned to a group (e.g. employees with depression) and which may trigger negative emotions against members of this group (e.g. fear, anger, frustration). These negative emotions lead to the third behavioral level, discrimination. Employees who suffer from depression may had prejudice against people with depression before and are aware of the prejudices of others [29]. This knowledge can lead to the anticipation and the fear of becoming a victim of stigmatization and social discrimination. In order to increase motivation to implement learned prevention-strategies and thus maintain an employee’s work ability after a depressive episode, it is necessary to reduce the anticipation and fear of stigmatization. To reduce stigmatization in the work context, different strategies can be applied; these can take place at the individual and the group level. There are findings from social-psychological research for reducing prejudice and discrimination. An individual strategy is, for example, perspective-taking. If colleagues and supervisors take the perspective of the affected employee (e.g. “Imagine, you return to work after a depressive episode. Which behavior would you like your colleagues to behave like?”), then this promotes empathy towards the employee with depression and reduces the negative evaluation [30]. The reduction of negative evaluation leads to less stigmatization. A further strategy at an individual level is a cooperation between the psychotherapist of the affected employee and the occupational physician. Nassri et al. [31] report that occupational physicians see a necessity for interdisciplinary cooperation with psychotherapists in the care of employees with depression. Occupational physicians have information about organizational structures at the work place and psychotherapists about the mental health status and the AS of the affected employee. A cooperation of occupational physicians and the psychotherapists can reduce the AS of employees who return to work after a depressive episode. At group level, it is important to understand that groups are characterized by their specific attributes. Based on these attributes, individuals are categorized as a group member or not. It is useful to design the attributes, with which employees define their company, in a way that the definition includes employees with depression. Managers report less stigmatization of depressed employees when the company has a clear approach to mental health promotion [32]. By contrast, managers report more stigmatization if the company has no support policies to maintain mental health. This means if mental health promotion is part of the norm and definition of the company, less stigmatization should take place. Furthermore, contradictory to the intention behavior gap we find reduced implementation behavior for employees reporting high AS and low intention. A reason for this may be the study design. As this is a cross-sectional study, no causal conclusions can be drawn from the results. It is possible that AS influences the intention and thus also the probability of implementing learned prevention-strategies. Nevertheless, in our study no association was found between both types of stigmatization with intention.

Results of the complementary research questions show that regarding both types of stigmatization, AS and ES, it is important whether only one or more than one depressive episode has been experienced so far. Participants with a recurrent depressive episode anticipate more stigmatization and report more experiences of stigmatization. A possible explanation for the differences in AS could be previous experiences of the participants. If a participant has already experienced stigmatization in a previous episode, this could lead to more AS in further depressive episodes. Related to the differences ES, the experience of stigmatization in an earlier depressive episode may result in a higher sensitivity for the perception of possible stigmatization in following episodes. In fact, our results show that depression and stigmatization are positively associated.

Very strict inclusion criteria were used in this study. This is a strength of the study, because it reduced biases from possible influencing factors. For example, only participants without an acute depressive episode were included in the analysis. Depressed patients perceive stigmatization as being stronger compared to people who are not depressed [21]. In our sample as well, participants with depression report a stronger stigmatization than participants without depression (see Additional file 1: Table S1). Only employees without a current depressive episode who returned to their work after sick leave because of depression were recruited. However, these strict inclusion criteria potentially result in a very small study population. These small study sample may be a source of reduced statistical power. Nevertheless, statistical power analyses confirm an adequate power of .81 for the moderation analysis and of .91 for the ANOVA [33]. Additionally, the bootstrapping analysis supports the results from the moderation analysis, because the 95% CI does not include zero. This approach strengthens our results. Further, the recruitment of the study population may cause a selection bias, because the participants had a free choice of participation in a study about “depression and work”. It is possible, that only employees participated who have problems in their work place due to their sick leave because of depression. This could cause a trend in the results. Another limitation of this study is that it was not ask for a specific intervention or prevention-strategy. Instead, participants were openly asked about prevention-strategies they had learned. The findings would be more precise with a targeted questioning of specific strategies, but the findings of this study can be better generalized because we used open questions to get information on the learned strategies. Furthermore, it should be discussed that intention to implement the learned prevention strategies was asked retrospectively. This can result in a recall bias so that the success of the implementation and effectiveness of the implemented prevention strategies influence the perception of the intention they had to implement the strategies before coming back to the work-place. A longitudinal study design with a measurement occasion before the participants go back to work and measurement occasions when they are back at work would be helpful to reduce this recall bias. Future studies should use a longitudinal study design to investigate the association between intention and implementation of prevention strategies. A last point of discussion is that stigmatization by colleagues and supervisors were asked within the same items. This makes it impossible to differentiate between the stigmatization by colleagues and supervisors. It is possible that stigmatization by colleagues has other effects than stigmatization by supervisors. However, to the best of author’s knowledge, there are no scientific findings on this in the literature. In future studies, a separate consideration of stigmatization by colleagues and supervisors should be carried out.

## Conclusions

This pilot study give first hints that AS moderates the association of intention and the behavior regarding the implementation of learned prevention-strategies in the workplace of employees returning from depressive episodes. In addition, participants with recurrent depressive episode seem to anticipate more stigmatization than participants after their first episode. Future studies should investigate the causality of the described association with a longitudinal study design. The aim in the organizational practice should be to create an understanding of depression and to implement norms in companies that include mentally ill employees (e.g. with depression) in cooperation with occupational physicians. The anticipation of stigmatization should be already addressed and reduced in the clinical setting, in order to motivate affected employees to implement learned prevention-strategies and thus protect themselves from a recurrence and maintain their work ability.

## Supplementary information

**Additional file 1:** **Table S1.** Mean differences in the stigmatization variables in participants without and with depression (t-test).

## Data Availability

The datasets analyzed during the current study are not publicly available. Participant- and health-related raw data are not allowed to be shared, but anonymized data of the scales are available from the corresponding author upon reasonable request.

## Abbreviations

ANOVA: Analysis of variance
AS: Anticipated stigmatization
B: Unstandardized Regression coefficient
CI: Confidence interval
df: degrees of freedom
DSM-IV: Diagnostic and statistical manual of mental disorders, 4th edition
ES: Experienced stigmatization
F: F-value
H: Hypothesis
ICD-10: International classification of diseases, 10th revision
ISE: Inventar subjektiver Stigmaerfahrungen (Inventory of subjective stigma experiences)
M: Mean
N/n: Number of participants
p: *p*-value (significance)
PHQ: Patient health questionnaire
PHQ-D: German version of the PHQ
R^2^: Explained variance
RQ: Research question
SD: Standard deviation
SE: Standard error
t: t-value
β: Standardized regression coefficient
η^2^: Eta square (explained variance)
