# Supplementary material for: Does stigmatization moderate the association between intention and implementation of learned prevention-strategies at work after a depressive episode? – a cross-sectional pilot study
Source: J Occup Med Toxicol. 2019 Nov 5;14:26. doi: 10.1186/s12995-019-0246-9 (PMC6833174; doi:10.1186/s12995-019-0246-9)
Supplement: Supplementary file 1 — Additional file 1: Table S1. Mean differences in the stigmatization variables in participants without and with depression (t-test). [file 12995_2019_246_MOESM1_ESM.docx]

Supporting information:

**Does stigmatization moderate the association between intention and implementation of learned prevention-strategies at work after a depressive episode? – A cross-sectional survey**

Petra Maria Gaum^1^, Franziska Brey^1^, Thomas Kraus^1^, Jessica Lang^1^

^1^Institute for Occupational, Social and Environmental Medicine, RWTH Aachen University, Germany

Corresponding author:

Dr. rer. medic. Petra M. Gaum

University Hospital RWTH Aachen

Institute for Occupational, Social and Environmental Medicine

Pauwelsstraße 30

52074 Aachen, Germany

Email: pgaum@ukaachen.de

Web: www.arbeitsmedizin.ukaachen.de

Phone: (+49) 241 80 89 040

Fax: (+49) 241 80 82 587

This supportive information consists of one table. The table reports differences in participants without and with current depressive episode according to anticipated and experienced stigmatization (table S-2). Stigmatization refers to both, stigmatization by colleagues and supervisors.

Table S-1: Mean differences in the stigmatization variables in participants without and with depression (t-test)

| Anticipated stigmatization | *N* | *M* | *SD* | *t* | *df* | *p* |
| --- | --- | --- | --- | --- | --- | --- |
| Without depression | 112 | 2.6 | 1.1 | -2.6 | 184 | .009 |
| With depression | 74 | 3.0 | 1.1 |  |  |  |
| Experienced stigmatization |  |  |  |  |  |  |
| Without depression | 112 | 1.8 | 1.1 | -2.7 | 141.0 | .009 |
| With depression | 74 | 2.3 | 1.2 |  |  |  |

Notes: N = number of participants, M = mean, SD = standard deviation, t = t-value, df = degrees of freedom, p = p-value (significance).
